# Association of lifestyle behaviours with self-esteem through health-related quality of life in Spanish adolescents

**DOI:** 10.1007/s00431-017-2886-z

**Published:** 2017-03-06

**Authors:** Emily Knox, Jose Joaquin Muros

**Affiliations:** 1 School of Health Sciences, University of Nottingham, Queens Medical Centre, Nottingham, UK; 2 Department of Nutrition and Food Science, University of Granada, Granada, Spain

**Keywords:** Mediterranean diet, Physical activity, Self-esteem, Health-related quality of life, Adolescents

## Abstract

The present research examined the association of Mediterranean diet adherence and physical activity with self-esteem through five components of health-related quality of life. Data were collected from 456 adolescents attending one of five schools in Granada, Spain using a cluster-randomised design. Participants completed questionnaires on Mediterranean diet adherence, physical activity, self-esteem and health-related quality of life (HRQoL). Models were constructed to identify associations between Mediterranean diet adherence and physical activity on self-esteem. Mediational analysis using bootstrapped confidence intervals examined possible mediation by five components of HRQoL. Mediterranean diet adherence and physical activity engagement were associated with four components of HRQoL: more positive physical wellbeing, psychological wellbeing, family relationships and autonomy support and perceptions of the school environment. Both lifestyle behaviours were positively associated with self-esteem. Both relationships were mediated through positive psychological wellbeing and perceptions of the school environment. Physical wellbeing was also a mediator of the relationship between physical activity and self-esteem.

*Conclusion*: Interventions promoting Mediterranean diets or physical activity to adolescents may facilitate improvements in self-esteem in addition to wider health benefits previously identified. Approaches within such interventions targeting improvements in physical wellbeing, psychological wellbeing and positive perceptions of the school environment may improve their efficacy.

**Table:** 

**What is Known:** • *It is known that engagement in lifestyle behaviours such as physical activity is positively linked with psychological health.* • *Whilst its consumption is declining, the Mediterranean diet is nutritionally recommended and remains popular in parts of Greece, Southern Italy and Spain. Research into Mediterranean diet adherence and psychological health is lacking.*
**What is New:** • *The present research furthers this knowledge by examining potential mechanisms through which two lifestyle behaviours (physical activity and following a Mediterranean diet) may be associated with self-esteem.* • *Implications for the promotion of positive mental health in young people. Mediterranean diet and physical activity were positively associated with self-efficacy via positive psychological self-concept and perceptions of the school environment. These novel findings can contribute to the development of more efficacious interventions targeting positive self-esteem in young people.*

## Introduction

Adolescence is the period in human growth and development that occurs after childhood and before adulthood, between the ages of 10 and 19 years [21, 22]. It can be an especially challenging period of the life course as rapid cognitive, physical, psychological and emotional changes take place which impinge on health and wellbeing [3]. It is estimated that around 20% of Spanish individuals will encounter some form of mental health disorder at some point during their adolescence [17]. For these reasons the mental health of young people is a national priority in Spain [17] and across Europe [38]. Low-self-esteem is associated with lower academic achievement and anxiety, depression and eating disorders [1]. On the other hand, high self-esteem has been associated with good mental health and developing and protecting self-esteem has been advocated as a key approach in prevention and mental health promotion [15]. The school offers a potential setting for intervention as aspects such as school peers have been found to have a strong impact on self-esteem during adolescence [32].

Engaging in healthy lifestyle behaviours, such as physical activity or following a healthy diet, is associated with positive self-esteem and mental health. For instance, physical activity has been associated with positive self-esteem in adolescents [2, 19, 20, 28]. Adhering to a Mediterranean diet, characterised by high consumption of olive oil, fruits, vegetables, whole grains, moderate to high consumption of fish, moderate consumption of milk and dairy products and low consumption of meat and meat products [35], is suggested to also relate to more positive mental health outcomes [11]. Despite this, only around 22.8% of Spanish adolescents (aged 11–17 years old) meet physical activity guidelines [39]. In addition, adherence to a Mediterranean diet is also low [30]. In the last 40 years, noticeable modifications to the dietary habits of adolescents have been observed in the Mediterranean countries, resulting in an increase in the consumption of processed food, refined sugar, saturated fats and cholesterol [29]. These two behaviours (physical activity and MD adherence) therefore offer a potential opportunity for targeting improved mental health of adolescents.

Improved understanding of the mechanisms through which physical activity or Mediterranean diet adherence can improve self-esteem would facilitate the development of more effective interventions. In a recent study by Breslin and colleagues [4], positive associations were identified between physical activity and aspects of health-related quality of life in 9- to 11-year-old children. The authors called for greater consideration of the specific relationships between wellbeing and physical activity when conducting interventions with children. The present research aims to identify the channels through which self-esteem is most likely to be enhanced by physical activity. We will explore whether physical activity and adherence to a Mediterranean diet are associated with self-esteem through five different components of health-related wellbeing (physical, psychological, family relationships and autonomy, peer relationships and social and the school environment). The findings will inform the development of more effective interventions within similar adolescent populations.

## Methods

### Subjects

Participants were recruited from their schools to participate in this cross-sectional research. Between 2014 and 2015, there were 20,929 adolescents enrolled at schools across Granada. The study involved 456 adolescents aged between 11 and 14 years, of which 235 were girls and 221 boys. Demographic characteristics of the study sample are provided in Table 1. Data were collected between March and May in 2014. Power analysis suggested that the study required a minimum sample of 378 adolescent to achieve sufficient power with a 95% confidence interval (*α* 0.05; *β* 0.2). Five of the 55 public schools in the city centre of Granada (Spain) were randomly selected to participate in this research. All participating schools were in a medium-high socioeconomic area based on information contained in the educational project of the centre or school. All adolescents from the five schools aged between 11 and 14 years (*N* = 511) were invited to take part in this study. The sample was recruited from five schools in Granada (Spain) in a cluster-randomised design. Five hundred and eleven adolescents were selected and invited to take part in this study. Of these, 480 agreed to participate and written informed consent was received from their parent or guardian. Twenty-four adolescents were excluded for failing to complete some element of testing, or because they failed to attend class on their testing day. Both the adolescent and their parents or guardians were informed of the objectives and methods of the study and told that they could withdraw at any time. Participants were instructed on how to fill out the questionnaires and how to conduct the tests. All tests were conducted during participants’ physical education lesson in school time. No incentives were provided to adolescents or parents. A research assistant was also on hand to provide guidance on the completion of questionnaires and conduct physical testing. Ethical approval was granted by the ethics committee of the University of Granada. Ethical principles of the Declaration of Helsinki for medical research were adhered to.

**Table 1:** Baseline characteristics of the study sample

	Sample (*N* = 456)
Age (years)	12.57 ± 1.17
Gender (% male)	51.5%
BMI (kg/m^2^)	19.75 ± 3.85
Physical activity (score)	2.92 ± 0.64
Mediterranean diet adherence (score)	7.87 ± 2.08
Self-esteem (score)	33.13 ± 5.37
Physical wellbeing (score)	52.79 ± 12.11
Psychological wellbeing (score)	52.70 ± 11.08
Family relationships and autonomy support (score)	50.57 ± 9.60
Social relationships and peer pressure (score)	54.86 ± 10.16
School environment (score)	54.34 ± 10.04

Data shown as mean ± SD. Mediterranean diet adherence: ≥8, good; 4–7, average; ≤3, poor

*BMI* body mass index

### Health-related quality of life

To assess health-related quality of life (HRQoL), we used the KIDSCREEN-27 questionnaire [34]. This internationally validated instrument [25] has been applied in populations of healthy and chronically ill children and adolescents aged from 8 to 18 years. The KIDSREEN-27 consists of 27 items relating to five components (physical wellbeing, psychological wellbeing, autonomy and relationship with parents, social support and peers and school environment). Internal consistency of the subscales was between 0.81 and 0.84, and the test-retest reliability of the subscales ranged from 0.61 to 0.74 [20]. Higher scores indicate higher HRQoL.

### Anthropometric measurement

Height and weight were measured following the protocols established by the International Society for the Advancement of Kinanthropometry [31] using a stadiometer (GPM, Seritex, Inc., Carlstadt, NJ; ±1 mm accuracy) and an electronic scale (model 707, Seca Corporation, Columbia, MD; ±50 g accuracy); body mass index (BMI) was calculated as weight divided by height squared (kg/m^2^).

### Physical activity

Physical activity levels were evaluated using the Physical Activity Questionnaire for Older Children (PAQ-C). The questionnaire provides a general measure of physical activity for 8- to 20-year-olds. The PAQ-C is a self-administrated questionnaire consisting of nine items rated on a five-point scale. A higher score indicates more active children. Respondents are asked to recall the frequency and type of physical activity they have engaged in on each of the 7 days prior to completing the questionnaire. Validation studies have found the PAQ-C to be highly reliable [27].

### Adherence to the Mediterranean diet

Adherence to the Mediterranean diet was assessed using the Evaluation of the Mediterranean Diet Quality Index (KIDMED) [30] which was created to estimate adherence to the Mediterranean diet in children and young adults. The test comprises 16 dichotomous items (yes/no) of which 12 items describe behaviours consistent with the Mediterranean diet, e.g. “Do you use olive oil at home?” and four items describe behaviours inconsistent with the Mediterranean diet, e.g. “Do you consume sweets and candy several times every day?”. Affirmative answers to Mediterranean diet consistent and inconsistent behaviours were scored +1 and −1 respectively, giving a maximum possible score of 12.

### Self-esteem

Self-esteem was evaluated using the Rosenberg self-esteem scale [26]. This self-report questionnaire consists of 10 items rated on a four-point Likert scale, ranging from 1 (strongly disagree) to 4 (strongly agree). Five items are positively worded (e.g. “On the whole, I am satisfied with myself”), and five are negatively worded (“Sometimes I feel really useless”). A minimum score of 10 points and a maximum score of 40 points are possible, with higher scores indicating higher self-esteem. The scale was translated and validated with Spanish students [16], showing satisfactory internal consistency (0.85 to 0.88) and test-retest reliability (0.84).

### Statistical analysis

Path analysis using SPSS 22.0 was employed to evaluate whether the five components of HRQoL explained (i.e. mediated) the relationships between the independent variables (physical activity and adherence to the Mediterranean diet) and self-esteem. One model was created for physical activity and one model was created for adherence to Mediterranean diet. Both models were adjusted to control for BMI and gender. Bootstrapping was also applied to the models in order to improve statistical rigour. Bootstrapping analyses were conducted following the method of Preacher and Hayes [23] for estimating indirect effects in simple mediation models. To establish mediation using this method, four paths should be created and analysed. The first path is the simple effect of the independent variable on self-esteem (path *a*). The second path is the effect of the independent variable on the proposed mediator (the five components of HRQoL [path *b*]). The third path is the effect of the proposed mediator on self-esteem (path *c*) The final path (path *c*′) is the direct effect of the independent variable on self-esteem, controlling for paths *a* and *b*. Bootstrapped estimates of paths *a* (independent variable → mediator), *b* (mediator → self-esteem), *c*′ (direct effect of independent variable → self-esteem) and *c* paths (total effect of independent variable → self-esteem) were performed. As advised by Hayes [10], a causal steps approach was not used and the indirect effect was evaluated even when path *c* was non-significant. Bootstrapped estimates of path *c*′ were performed to test the model by which the predictor has no effect in the criterion when the mediator is controlled (i.e. moderation). Finally, the true indirect effect for the mediation models was tested through bootstrapped estimates of the product of paths *a* and *b* (a*b). Statistical significance for each path tested was established when zero did not lie between the 95% bootstrapped confidence interval, with 1000 bootstrap resamples.

## Results

### Mediterranean diet and self-esteem

**Fig. 1:**
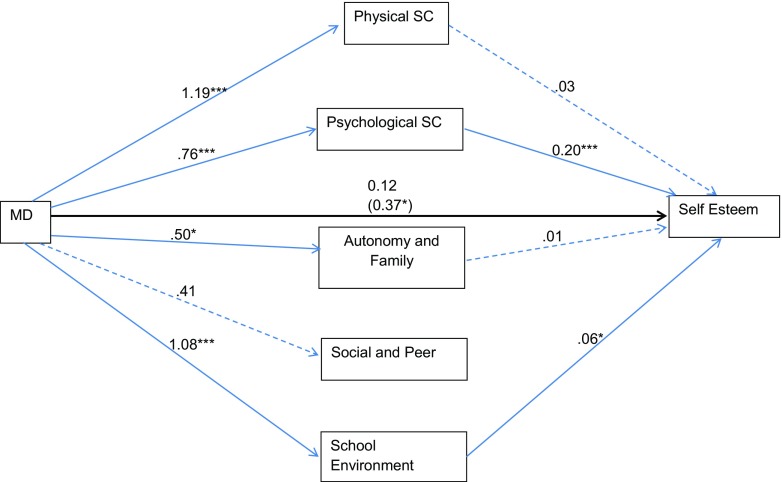
Mediational analysis of the influence of adherence to the Mediterranean diet (MD) on self-esteem through the five components of wellbeing. The *solid bold line* shows the direct effect of MD on self-esteem (path *c*′). *Solid light lines* show significant effects of the predictor (MD) on the mediator variables (HRQoL) (path *a*) and the significant effects of the mediator variables on self-esteem (path *c*). *Dashed lines* show non-significant effects

The first indirect effect model used adherence to the Mediterranean diet as the independent variable and the five components of subjective wellbeing (physical, psychological, family relationships and autonomy support, social relationships and peer pressure and school environment) as potential mediators. Results from this set of bootstrapped estimates are provided in Fig. 1 and described below.

Analyses suggested that Mediterranean diet was directly associated with self-esteem, i.e. when any confounding influence of the potential mediators was not considered (path *c*; *β* = 0.37, SE = 0.15, *p* < 0.05). Path *c*′, which examined the relationship of Mediterranean diet through the mediating variables, was non-significant (*β* = 0.12, SE = 0.13, *p* = 0.35), suggesting that the influence of following a Mediterranean diet on self-esteem was not mediated by all five components of subjective wellbeing. Values for each component were therefore scrutinised separately. Mediterranean diet was not associated with social relationships and peer pressure (path *a*; *β* = 0.41, SE = 0.25, *p* = 0.11). Physical self-concept and family relationships and autonomy support were then examined as mediator variables. The relationship between Mediterranean diet and each of these variables was significant (paths *a*; *β* = 1.19, SE = 0.29, *p* < 0.001; *β* = 0.50, SE = 0.23, *p* < 0.05). However, the relationship between these variables and self-esteem was not significant (path *b*), and so these variables were not explored further.

Mediterranean diet was significantly associated with psychological self-concept (path *a*; *β* = 0.76, SE = 0.28, *p* < 0.01). Further, psychological self-concept was associated with self-esteem (path *b*; *β* = 0.20, SE = 0.0.03, *p* < 0.001). Thus, the indirect effects were then tested by examining bootstrapped estimates of path a*b. Examination of confidence intervals suggested that psychological self-concept mediated the association between Mediterranean diet and self-esteem, since zero was not included (*β* = 0.15, SE = 0.06, CI = 0.04 to 0.30). Examination of the standardised effect (*β* = 0.06, SE = 0.02, SE = 0.02 to 0.11) and ratio of indirect to total effect (*β* = 1.25, SE = 22.38) produced the same conclusions.

Mediterranean diet was significantly associated with perceptions of the school environment (path *a*; *β* = 1.08, SE = 0.23, *p* < 0.001). Further, perception of the school environment was associated with self-esteem (path *b*; *β* = 0.06, SE = 0.03, *p* < 0.05). The indirect effects were also then tested. Examination of bootstrapped confidence intervals of path a*b suggested that the school environment mediated the association between Mediterranean diet and self-esteem, since zero was not included (*β* = 0.06, SE = 0.03, CI = 0.01 to 0.15). Examination of the standardised effect (*β* = 0.02, SE = 0.01, CI = 0.01 to 0.05) and ratio of indirect to total effect (*β* = 0.52, SE = 10.73) produced the same conclusions.

### Physical activity and self-esteem

**Fig. 2:**
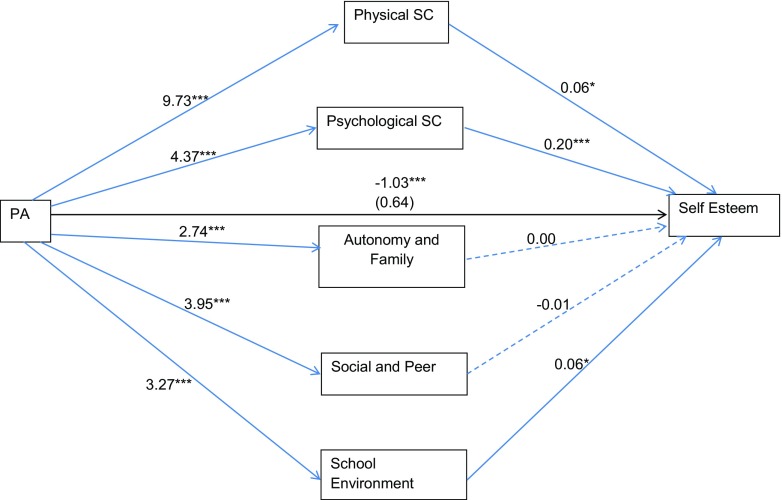
Mediational analysis of the influence of physical activity engagement on self-esteem through the five components of wellbeing

The second indirect effect model used engagement with physical activity as the independent variable and the five components of subjective wellbeing (physical, psychological, family relationships and autonomy support, social relationships and peer pressure and school environment) as potential mediators. Results from this set of bootstrapped estimates are provided in Fig. 2 and described below.

Analyses suggested that physical activity was not directly associated with self-esteem, when any influence of the potential mediating variables was not considered (path *c*; *β* = 0.64, SE = 0.49, *p* = 0.19). Path *c*′ (association of physical activity on self-esteem, when the potential mediating variables were considered) was significant (*β* = −1.03, SE = 0.48, *p* < 0.05), suggesting that physical activity did exhibit a relationship with self-esteem through the five components of subjective wellbeing. These mediation effects were further investigated. Physical activity was related with family relationships and autonomy support and social relationships and peer pressure (path *a*; *β* = 3.95, SE = 0.77, *p* < 0.001; *β* = 3.27, SE = 0.75, *p* < 0.001). However, none of these components of HRQoL were related to self-esteem (path *b*), and so these variables were not explored further.

Physical activity was related with physical self-concept (path *a*) (*β* = 9.73, SE = 0.86, *p* < 0.001). Further, physical self-concept was associated with self-esteem (path *b*; *β* = 0.06, SE = 0.03, *p* < 0.05). Bootstrapped confidence intervals of the indirect effects were then examined. Path a*b (*β* = 0.61, SE = 0.28, CI = 0.06 to 1.14) was significant as confidence intervals did not include zero, suggesting that physical self-concept mediated the relationship between physical activity and self-esteem. Examination of the standardised effect (*β* = 0.07, SE = 0.03, CI = 0.01 to 0.13) and ratio of indirect to total effect (*β* = −0.59, SE = 13.72) produced the same conclusions.

Physical activity was associated with psychological self-concept (path *a*; *β* = 4.37, SE = 0.82, *p* < 0.001). Further, psychological self-concept was associated with self-esteem (path *b*; *β* = 0.20, SE = 0.03, *p* < 0.001). Path a*b (*β* = 0.87, SE = 0.21, CI = 0.54 to 1.38) was significant suggesting that psychological self-concept also mediated the relationship between physical activity and self-esteem. Examination of the standardised effect (*β* = 0.10, SE = 0.02, CI = 0.06 to 0.15) and ratio of indirect to total effect (*β* = −0.85, SE = 54.51) produced the same conclusions.

Physical activity was associated with perceptions of the school environment (path *a*; *β* = 3.27, SE = 0.75, *p* < 0.001). Further, the school environment was associated with self-esteem (path *b*; *β* = 0.06, SE = 0.03, *p* < 0.05). Path a*b (*β* = 0.21, SE = 0.10, CI = 0.04 to 0.45) was also significant, suggesting that the school environment mediated the relationship between physical activity and self-esteem. Examination of the standardised effect (*β* = 0.02, SE = 0.01, CI = 0.01 to 0.05) and ratio of indirect to total effect (*β* = −0.20, SE = 10.06) produced the same conclusions.

## Discussion

Results from the present study suggest that adolescents who follow a Mediterranean diet tend to hold more positive perceptions of their physical wellbeing, psychological wellbeing, autonomy support and family relationships and of their school environment, regardless of their BMI or gender. Further, these adolescents also exhibit more positive self-esteem, and this appears to be partly attributable to the influence of following a Mediterranean diet on their psychological wellbeing and perceived school environment.

A recent study [37] identified a number of psychopathological benefits to be associated with Mediterranean diet adherence in Spanish school-aged children. This included reduced risk of depression or suffering from an eating disorder and low anxiety. The present study is the first to demonstrate the association of Mediterranean diet adherence with self-esteem through improved psychological wellbeing in adolescents. Georgiadis et al. [9] conducted cluster analyses according to self-esteem theory on a Greek sample of dieters. Worryingly, less than 30% demonstrated an adaptive psychological profile characterised by high self-esteem and less controlling diets. The Mediterranean diet has demonstrated vast benefits to health [6, 35, 36]. Further, adolescents adhering to a Mediterranean diet in our sample and throughout Spain as part of their regular lifestyle may be protected from the negative psychological aspects of restrictive dieting [9]. Crichton and colleagues [6] have also uncovered that a Mediterranean style diet is related with improved psychological functioning in Australian adults even when adherence was not high. Eating foods consistent with the Mediterranean diet could therefore be important across the lifespan. Further research is required to uncover the mechanism through which the Mediterranean diet might exert this influence.

The present study also suggests that positive perceptions of the school environment positively impacts self-esteem in active adolescents who adhere to a Mediterranean diet. Previous research has linked Mediterranean diet adherence to better academic performance of Mediterranean children [8, 36], and academic performance has been linked to self-esteem [24]. It is possible that the adolescents in the present sample had higher academic attainment which led to them more positively perceiving their school environment. As academic attainment of the present sample was not measured, further research including observational studies may be useful to illuminate the reasons for these more positive perceptions of the school environment.

The second part of this research revealed that physically active adolescents also tended to respond more positively to all five components of wellbeing. The associations with physical activity were much stronger than those for Mediterranean diet adherence. Further, physical activity was positively associated with self-esteem through positive associations with physical wellbeing, psychological wellbeing and perceptions of the school environment.

The relationship between physical activity and physical and psychological wellbeing has been explored in numerous different populations [14, 18]. Strauss and colleagues [33] have also identified the importance of physical activity to the development of self-esteem in 10- to 16-year-olds. This is the first study to identify these constructs as mediators of self-esteem in Spanish adolescents. Moreno et al. [18] reported that physical activity positively influenced self-esteem and physical wellbeing specifically in a sample of 2332 students aged 9–23 years. These authors also identified gender and age differences. Other research has suggested that overweight adolescents may especially benefit from engaging in physical activity in terms of self-esteem [28]. The present research identified mediating effects of physical wellbeing on self-esteem. Many adolescents experience physical changes which can lower their self-esteem [13], and it is possible that physically active adolescents possess a healthy body image of themselves simply because they are active [12]. The present findings indicate that physical activity could be especially critical at this time to maintain positive physical wellbeing. Further, psychological wellbeing may be especially important for maintaining high levels of motivation to be active [7]. Interventions to increase physical activity levels of adolescents should, therefore, seek to raise adolescent’s self-referenced perceptions of physical and psychological wellbeing to improve their self-esteem.

Positive perceptions of the school environment also mediated the relationship between physical activity and self-esteem. Previous research has suggested that engagement in physical activity mediates perceptions of school-related stress with more active children reporting less felt stress [5]. The more positive perceptions of the school environment of active adolescents may be at least partly explained by their experience of less stress. It is also possible that these adolescents perceived more opportunities to practice physical activity at school. This reinforces suggestions that schools should be utilised as the setting of physical activity interventions.

### Limitations

Conclusions from the present research should be interpreted in light of a number of limitations. The research design was cross-sectional and so inferences around causality cannot be made. Further, self-report methods were employed which introduces possible measurement error. However, as both the IPAQ-C and the KIDMED have previously demonstrated high validity and reliability in this population, we believe that this should have little impact on the conclusions made. Further, interactions between physical activity behaviour and dietary habits could have a further influence on self-esteem which was not addressed in the present study. Future research could aim to do so. It would also be interesting to compare the present population with those from other areas of Spain. Given the findings relating to the perception of the school environment, it could be particularly interesting to examine rural areas, where the school environment is likely to be very different to that found in a city. Despite these limitations, this is the first study, to our knowledge, to analyse associations between Mediterranean diet adherence and physical activity on self-esteem through HRQoL in adolescents.

## Conclusions

The present study suggests that adolescents who follow a Mediterranean diet or who engage in more physical activity exhibit higher self-esteem. The original contribution of this research is the finding that both of these behaviours may relate to self-esteem through positive psychological self-concept and perceptions of the school environment. This has important implications for parents, teachers, youth workers, policy-makers and other professionals with a responsibility to protect the psychological health of young people. One approach might be to encourage schools to be the settings of behavioural interventions, either through provision of opportunities to engage in the behaviours or provision of information promoting them. Other approaches could be to provide educational sessions within Mediterranean diet or physical activity interventions which encourage a positive body image. However, it is not only the schools who must shoulder responsibility. Policy-makers should reinvigorate efforts to educate parents and their children about the benefits of physical activity and the Mediterranean diet and direct resources to aid provision in schools. Where possible, youth workers should seek to offer opportunities to experience both, i.e. through cooking workshops or taster classes, whilst paediatricians should be able to both educate patients and signpost to community-based opportunities. Further studies are required to identify how healthy lifestyle approaches can have the most positive effect on self-esteem via psychological wellbeing, physical wellbeing and perceptions of the school environment.

## Abbreviations

BMI: Body mass index
HRQoL: Health-related quality of life
PAQ-C: Physical activity questionnaire for older children
